# Effect of Silver Diamine Fluoride on Bacterial Biofilms—A Review including In Vitro and In Vivo Studies

**DOI:** 10.3390/biomedicines11061641

**Published:** 2023-06-05

**Authors:** Hind Mubaraki, Navin Anand Ingle, Mohammad Abdul Baseer, Osamah M AlMugeiren, Sarah Mubaraki, Marco Cicciù, Giuseppe Minervini

**Affiliations:** 1 Preventive Dentistry Department, College of Dentistry, Riyadh Elm University, Riyadh 13244, Saudi Arabiao.almugeiren@riyadh.edu.sa (O.M.A.);; 2 Department of Biomedical and Surgical and Biomedical Sciences, Catania University, 95123 Catania, Italy; mcicciu@unime.it; 3 Multidisciplinary Department of Medical-Surgical and Dental Specialties, University of Campania, 80138 Naples, Italy

**Keywords:** bacteria, biofilms, caries, SDF, *Streptococcus*

## Abstract

Caries/carious lesions are a growing concern among the general population across the world, and different strategies are evolving to combat the bacterial invasion that resultantly leads to caries. In this systematic review, we are looking to analyse the role of silver diamine fluoride (SDF) on the growth of bacterial biofilms. The search strategy for the studies to be selected for the review was initiated by a search across multiple databases, which ultimately yielded 15 studies that were in accordance with our objectives. The reviewed articles indicate a very clear correlation between the usage of SDF and the decrease in bacterial biofilms, which are limited not just to one or two but multiple bacterial species. As shown by the events favoring SDF’s odds ratio of 3.59 (with a 95% confidence interval of 2.13 to 6.05), a risk ratio of 1.63 (1.32 to 2.00), and a risk difference of 0.28 (0.16 to 0.40), there was strong evidence that SDF is a successful treatment for reducing bacterial biofilms in dental practice. This study offers substantial proof that SDF works well to reduce bacterial biofilms in dentistry practices. We advise further investigation to examine the potential of SDF as a standard therapy choice for dental caries and related conditions given the obvious relationship between the use of SDF and the reduction in bacterial biofilms.

## 1. Introduction

Oral biofilms, also known as dental plaque, are complex microbial communities that form on the surfaces of teeth and other structures in the mouth [1,2,3,4]. These biofilms are composed of a variety of microorganisms, including bacteria, fungi, and viruses, and can vary in composition depending on factors such as diet, hygiene habits, and genetics [5]. The formation of oral biofilms begins with the attachment of bacteria to the surface of the tooth, known as the pellicle layer [6]. Over time, the biofilm can become thicker and more complex, with different layers of microorganisms forming different niches based on their specific needs and environmental preferences. For example, some bacteria may prefer to live in areas with more oxygen, while others may thrive in areas with less oxygen [7]. While they are a natural part of the mouth’s ecology, they can also pose a threat to oral health if left unchecked [8,9]. For example, certain types of bacteria in the biofilm can produce acids that damage tooth enamel and lead to cavities. Additionally, the build-up of plaque can lead to gum disease, which can cause tooth loss and other oral health problems [10,11].

The primary pathogens responsible for tooth decay come in various numbers and ratios and generate bacteria that make acids and bases [12,13,14]. Mutans streptococci (MS), one of the speculated acid-producing bacteria, was discovered to be crucial in the early stages of dental caries on both enamel and root surfaces after a comprehensive literature analysis [15,16,17]. This is true for several reasons, including the fact that MS is the species that is frequently isolated from a caries lesion, that it is highly acidogenic and aciduric, and that it can produce surface antigens and water-insoluble glucan, which promote bacterial adhesion to other bacteria and to the tooth’s surface. To break the closed loop of the process, a measure that can prevent the microorganisms that produce acid must be taken [18,19].

SDF is a unique and versatile liquid treatment that has garnered a great deal of attention in recent years [20]. SDF is a highly effective and cost-effective treatment for a wide range of dental issues, including tooth decay, gum disease, and oral infections [21,22]. In this article, we will explore the properties of SDF and the role it plays in modern dentistry [23,24]. SDF is a clear, odorless liquid that contains two main ingredients: silver and fluoride [25]. Silver is a powerful antimicrobial agent that has been used for centuries to treat various infections, while fluoride is a mineral that is known to strengthen tooth enamel and prevent cavities. When these two ingredients are combined, they create a powerful solution that is highly effective at killing bacteria and preventing the spread of infection [26].

One of the key advantages of SDF is its ability to penetrate deep into the tooth structure, where it can kill bacteria and prevent further decay [27]. This makes it an excellent choice for treating cavities and other dental issues that are difficult to reach with traditional dental tools. SDF is also highly effective at killing the bacteria that cause gum disease, which is a major contributor to tooth loss in adults [28].

In addition to its antimicrobial properties, SDF is also highly effective at preventing the spread of infection [29]. When applied to an infected tooth or gum tissue, it quickly kills the bacteria and prevents it from spreading to other parts of the mouth. This can be particularly important for patients who are at risk of developing oral infections due to weakened immune systems or other medical conditions [30].

Another important property of SDF is its ability to strengthen tooth enamel and prevent cavities [31]. Fluoride is a mineral that is known to bond with tooth enamel, making it stronger and more resistant to decay [31]. When applied to the teeth, SDF releases fluoride ions that bond with the enamel and help to prevent the development of cavities. This makes it an excellent choice for patients who are at risk of developing tooth decay due to poor oral hygiene or other factors [32]. Moreover, unlike traditional dental treatments, which can be painful and time-consuming, SDF can be applied quickly and easily in a single visit. This makes it an excellent choice for patients who are anxious about dental procedures or who have difficulty sitting still for long periods of time [33,34,35].

The creation of in vitro biofilm models has simplified the investigation of mouth biofilm today [36,37]. But this approach isn’t without several drawbacks [38]. An option is to use an in-situ biofilm model to study natural oral biofilms and various therapeutic approaches. Several gaps in the literature need to be addressed when examining the effect of SDF on bacterial biofilms. Firstly, there is a need for standardized protocols and methodologies to study the effect of SDF on biofilms. Varying experimental conditions, biofilm models, SDF concentrations, and application methods make it difficult to compare and synthesize findings across studies. Developing standardized protocols would enhance the reliability and reproducibility of research outcomes. Additionally, there is a lack of evidence regarding the long-term effects and durability of SDF on biofilms. Most studies focus on short-term effects, but understanding the persistence and long-term effectiveness of SDF in controlling biofilms is crucial. Furthermore, there is a need for more clinical research assessing the clinical outcomes and patient-centered effects of SDF on biofilms. Comparative studies that assess the effectiveness of SDF compared to other treatments for bacterial biofilms are also lacking. Comparative research would shed light on the relative efficacy, safety, and cost-effectiveness of SDF as a treatment option. Finally, further mechanistic understanding is needed to elucidate the underlying mechanisms of SDF’s action on bacterial biofilms. Investigating the interaction between SDF and biofilm components, as well as its impact on biofilm formation, maturation, and eradication, would enhance our understanding of its mode of action. Addressing these gaps in the literature might provide a comprehensive understanding of the effect of SDF on bacterial biofilms and guide its optimal clinical use. Therefore, this investigation aimed to systematically review and meta-analyse the available literature on the effect of SDF on bacterial biofilms. Secondarily, we also wanted to provide an overview of the existing evidence regarding the efficacy of SDF in reducing bacterial biofilms in dental practice. Additionally, the study aimed to evaluate the effectiveness of SDF compared to other biomarkers used in dental practice and to identify potential limitations or biases in the available literature.

## 2. Materials and Methods

### 2.1. Eligibility Criteria

All studies were assessed for eligibility according to the following participants, intervention, comparison, and outcomes (PICO) model:

(P) Participants in this systematic review are patients with bacterial biofilms. To include all relevant studies, the Boolean operator “OR” was used to combine different keywords related to bacterial biofilms, such as “biofilm”, “plaque” and “microbial colonies”.
(I) The intervention consisted of Silver Diamine Fluoride (SDF). To ensure that all relevant studies on SDF were included in the review, the Boolean operator “OR” was used to combine different variations of the keyword, such as “silver diamine fluoride” or “AgF”.
(C) The comparison consisted of control groups that compared SDF to other chemical compounds. To identify studies that compare SDF to other biomarkers/control groups, the Boolean operator “AND” was used to combine the intervention and comparison groups that were considered as interventions.
(O) The outcome measures the effect of SDF on bacterial biofilms. To identify studies that measure the effect of SDF on bacterial biofilms, the Boolean operator “AND” was used to combine the intervention and outcome keywords.

Only studies providing data at the end of the intervention were included. Exclusion criteria were as follows: (1) studies that did not investigate the effect of SDF on bacterial biofilms or microbial colonies; (2) studies that did not compare SDF to other biomarkers or control groups; (3) studies that did not report a statistical analysis of the evaluation of SDF; (4) studies that considered the effect of SDF but abstained from revealing the findings; (5) cross-over study design; (6) studies written in a language different from English; and (7) full-text unavailability (i.e., posters and conference abstracts).

### 2.2. Search Strategy

PubMed, Scopus, and Web of Science databases were systematically searched for articles published from the year 2013 until 2022, following the strategy described in Table 1. Furthermore, a manual search of the references of previous systematic reviews on a similar topic was conducted as well.

**Figure 1:**
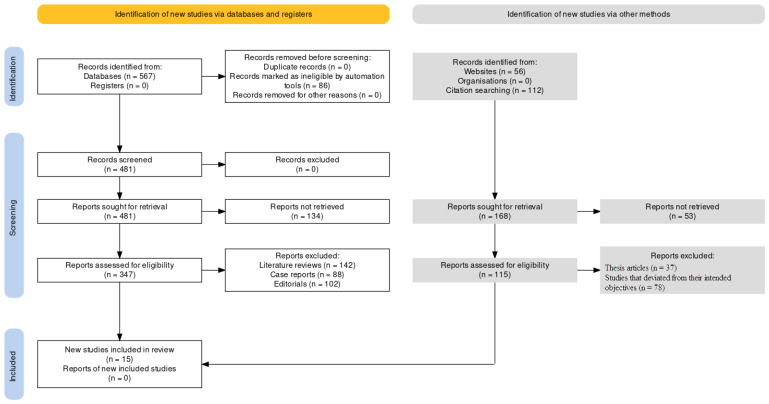
Representation of selection of articles through the PRISMA framework.

This systematic review with meta-analysis was conducted according to the guidance of Preferred Reporting Items for Systematic Reviews and Meta-Analyses (PRISMA) guidelines [39], Figure 1 and the *Cochrane Handbook for Systematic Reviews of Interventions* [40]. The systematic review protocol has been registered on the International Prospective Register of Systematic Reviews (PROSPERO) with acknowledgement of the receipt number 405877.

### 2.3. Data Extraction

Two reviewers (S.O. and S.C.) independently extracted data from the included studies using a customized data extraction on a Microsoft Excel sheet. In case of disagreement, the consensus was achieved through a third reviewer.

The following data were extracted: (1) first author; (2) publication year; (3) nationality; (4) type of study design; (5) type of compounds as intervention; (6) type of control (placebo or other); (7) population and the number of patients/biofilms included; (8) SDF’s efficacy as an outcome; and (9) main findings.

### 2.4. Quality Assessment

**Figure 2:**
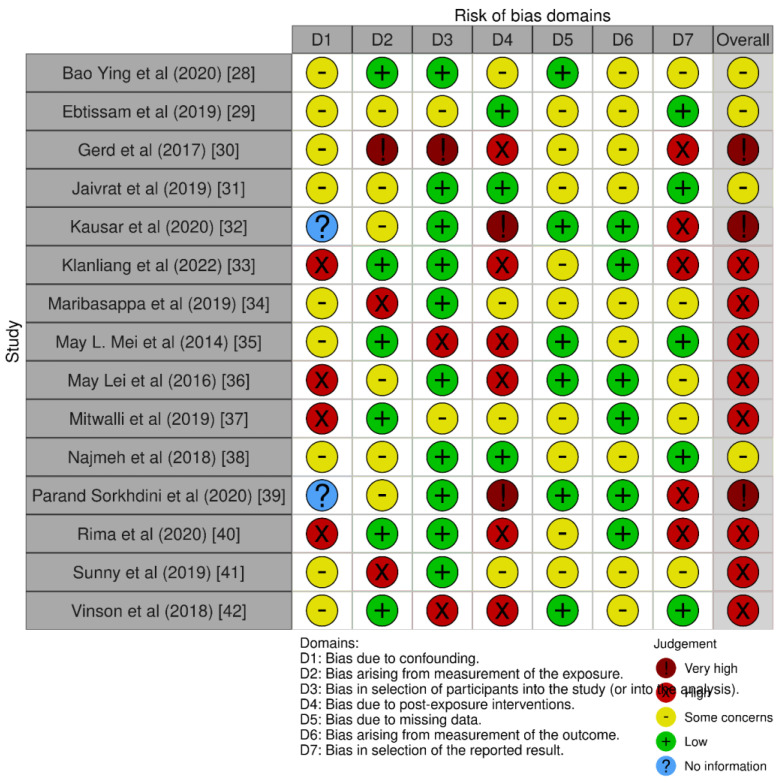
ROBINS-E tool for bias assessment for papers selected for the systematic review.

The ROBINS-E tool was used for the assessment of bias in the selected papers for our review (Figure 2). The tool is used to evaluate the risk of bias in cohort studies, case-control studies, and before–after studies, among others [41].

### 2.5. Statistical Analysis

Using the data extraction form that the reviewers had compiled in a single dataset, the statistical protocol for this study was initiated by entering the data into RevMan 5, choosing the random effects statistical model for the meta-analysis, calculating the odds ratio, risk ratio, or risk difference for each study and entering the data into the RevMan 5 software (IBM, version 5.4.1, New York, NY, USA), thereby generating a forest plot to graphically display the results of the meta-analysis, assessing the heterogeneity of the studies using the I^2^ statistic, interpreting the results of the meta-analysis and drawing conclusions about the effect of SDF on bacterial biofilms, conducting sensitivity analyses to examine the robustness of the meta-analysis results, interpreting and reporting the results of the sensitivity analyses (which included the forest plot, summary of the effect estimates, and interpretation of the heterogeneity and sensitivity analyses).

## 3. Results

After conducting an extensive search of online journals, a total of 207 documents were identified, out of which 103 papers were initially selected. After eliminating 47 similar or duplicate articles, 56 separate papers were available for further review. Subsequently, after reviewing the abstracts and titles of the submissions, a further 41 papers were excluded. Finally, 15 studies that met the inclusion and exclusion criteria were included in the systematic review and meta-analysis. These studies comprised eight in vitro studies, four case-control studies, and one in situ, ex situ, and ex vivo study each.

**Figure 3:**
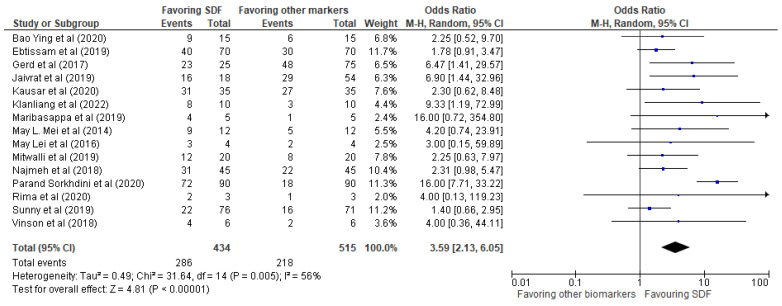
Odds ratio of events favoring SDF vs. other biomarkers/control groups.

**Figure 4:**
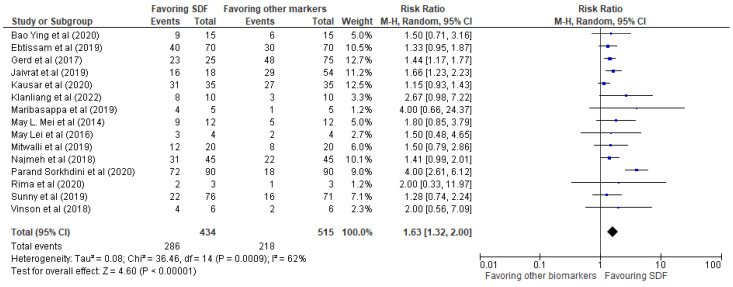
Risk ratio of events favoring SDF vs. other biomarkers/control groups.

**Figure 5:**
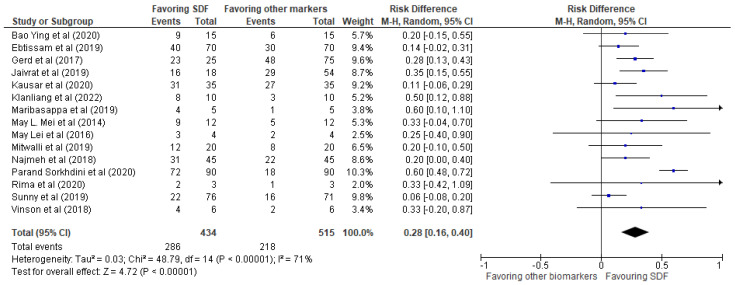
Risk difference of events favoring SDF vs. other biomarkers/control groups.

The results of the systematic review have been presented in Table 2, providing details of the 15 studies included in the review. The meta-analysis was performed using RevMan 5 software, and the results were presented in the form of forest plots depicting the odds ratio, risk ratio, and risk difference, as shown in Figure 3, Figure 4 and Figure 5, respectively.

Our investigation revealed a very positive impact of SDF compared to other biomarkers mentioned in the 15 studies. The events favoring SDF demonstrated an odds ratio of 3.59 (2.13, 6.05), a risk ratio of 1.63 (1.32, 2.00), and a risk difference of 0.28 (0.16, 0.40). These findings provide strong evidence that SDF is an effective treatment for reducing bacterial biofilms in dental practice.

The implications of these findings are significant for improving oral health outcomes, particularly for individuals who may not have access to traditional dental treatments or who may have difficulty receiving such treatments. The clear correlation between the usage of SDF and the decrease in bacterial biofilms makes it a potential standard treatment option for dental caries and related conditions. Therefore, further research is recommended to explore the potential of SDF in dental practice.

The statistical analysis displayed an odds ratio of 3.59 (2.13, 6.05) for events favoring SDF compared to other biomarkers/control groups in the selected studies as represented in Figure 3. The forest plot visualized the risk difference for each study included in the meta-analysis, which represented the difference in the proportion of events favoring SDF between the SDF and control groups. The analysis found heterogeneity between studies, with a Tau^2^ of 0.49 and a Chi^2^ of 31.64 with 14 degrees of freedom (*p* = 0.005) and an I^2^ of 56%. The significant *p*-value for Chi^2^ indicates that this heterogeneity was unlikely to have occurred by chance. The Z-statistic for the overall effect was 4.81 (*p* < 0.00001), indicating that the odds of events favoring SDF were significantly higher in the SDF group compared to the control group. In summary, the statistical analysis suggests that SDF is associated with a higher odd of events favoring SDF compared to other biomarkers/control groups, with some degree of heterogeneity between studies. Further research may be needed to better understand this variation.

The risk ratio of 1.63 (1.32, 2.00) is displayed in Figure 4 for events favoring SDF compared to other biomarkers/control groups in the selected studies. The forest plot displayed the risk difference for each study included in the meta-analysis, representing the difference in the proportion of events favoring SDF between the SDF and control groups. The analysis found heterogeneity between studies, with a Tau^2^ of 0.08 and a Chi^2^ of 36.46 with 14 degrees of freedom (*p* = 0.0009) and an I^2^ of 62%. The Z-statistic for the overall effect was 4.60 (*p* < 0.00001), indicating that the odds of events favoring SDF were significantly higher in the SDF group compared to the control group.

The representation of the risk difference in the form of a forest plot in Figure 5 displayed the individual studies included in the meta-analysis, the value of which was found to be 0.28 (0.16, 0.40). The results showed some heterogeneity, with a tau-squared value of 0.03 and a Chi^2^ value of 48.79 with 14 degrees of freedom, resulting in a *p*-value of less than 0.00001. This indicated significant heterogeneity in the results. The overall effect was statistically significant, with a Z-score of 4.72 and a *p*-value of less than 0.00001. The analysis indicated that SDF had a significant risk difference compared to other biomarkers/control groups, and while some heterogeneity was observed, the overall effect was statistically significant.

## 4. Discussion

The findings observed through the meta-analysis reveal important information about the effectiveness of SDF as a therapy for bacterial biofilms. The results of the review show that SDF, both alone and in contrast to other conventional antibacterial and antifungal substances frequently used in dentistry, has a very favorable effect on reducing bacterial biofilms. This is a significant finding because it implies that SDF may be a more effective treatment option for dental caries and related conditions, especially for people who might not have access to or struggle with conventional dental treatments. This study also emphasizes SDF’s promise as a standard treatment for dental caries and associated conditions. SDF may be a useful and efficient way to improve oral health outcomes, especially in places where access to conventional dental treatments is restricted. This is supported by the clear correlation between the use of SDF and the decline in bacterial biofilms across a variety of bacterial species. This comprehensive review and meta-analysis, in addition, offer crucial direction for further study in this field. The review’s conclusions suggest that additional study is required to fully investigate SDF’s potential as a remedy for bacterial biofilms and associated conditions. Studies examining the long-term benefits of SDF therapy and the possibility of combining SDF with other therapies to further enhance oral health outcomes may fall under this category.

In a clinical trial [53], a single SDF application reduced the burden of care for weak, dependent patients. An annual application of SDF varnish halted the active root carious lesions in nearly 90% of cases in one of the studies [44]. Therefore, it seems that SDF applied by an expert is effective in delaying the onset and development of root caries [54]. Nevertheless, there have also been claims that SDF treatment has disadvantages, including staining/discoloration [55,56,57], an inflammatory impact on the dentin–pulp complex, and a decrease in bond strength [58].

When a serious caries lesion has already developed, root caries cannot be successfully treated by simply controlling plaque and using fluoride treatments [59,60]. Because dentin’s decay and flaws have progressed, restorative materials must be used as a management strategy. The progression of tooth decay can be halted with SDF therapy, but it is possible that cavities will need to be filled with dental fillings. The restorative approach is regarded as a non-traumatic method that could provide patients with a sense of aesthetic gratification [61]. In order to effectively treat advanced root caries lesions, the focus has been placed on the use of restorative materials in combination with SDF administration [60].

Discoloration, one of the main problems that plagues SDF, has been the subject of numerous efforts. A study suggested following SDF usage with KI application to address the issue [62]. Another clinical study revealed that the use of KI did not, however, have a long-term impact on resolving the staining problem, and the discoloration eventually returned [44]. There have been other suggested remedies for staining, such as adding glutathione to SDF, which significantly lessened the coloring but left some pigmentation [63]. Other efforts have used different compounds with somewhat promising results [64,65], but more studies are needed in that regard.

AgNPs have garnered considerable scientific interest and significance owing to their distinct characteristics and broad-ranging applications in diverse scientific domains [66]. Their small size and large surface area-to-volume ratio contribute to heightened reactivity and unique physicochemical properties, rendering them highly versatile nanomaterials. A primary facet of the importance of silver nanoparticles lies in their remarkable antimicrobial efficacy [67]. They exhibit potent antimicrobial activity against a wide spectrum of microorganisms, encompassing bacteria, fungi, and viruses. This renders them prospective candidates for the development of antimicrobial agents, disinfectants, and coatings that impede the growth and dissemination of pathogens [66,67]. Their integration into healthcare settings, including wound dressings, medical devices, and antibacterial coatings, holds promise for mitigating the risk of infections and enhancing patient outcomes.

A couple of studies provide important insights into the synthesis and potential applications of AgNPs. In the first study [66], silver nanoparticles were synthesized using olive leaf extract as a reducing agent. The results demonstrate the feasibility of using natural extracts for the synthesis of AgNPs. This study highlights the green synthesis approach and the potential of synthesized AgNPs using olive leaf extract. In the second study [67], an aqueous extract obtained from chickpea leaves was used for the synthesis of AgNPs. The results reveal the successful synthesis of AgNPs with specific properties, including a maximum surface plasmon resonance wavelength, crystallite dimension, and size range. The inhibitory effect of AgNPs on food pathogen strains and yeast was evaluated, demonstrating high effectiveness at low concentrations against certain strains. The cytotoxic effects of AgNPs on cancerous and healthy cell lines were also investigated, showing no significant decrease in cell viability with increased AgNPs concentration. Overall, these studies [66,67] emphasize the importance of silver nanoparticles as versatile and potentially valuable nanomaterials. The green synthesis approach using natural extracts offers an eco-friendly and cost-effective method for their production. The synthesized AgNPs exhibit desirable characteristics such as specific size, shape, and inhibitory effects against pathogens. The findings support the exploration of AgNPs for various applications, including antimicrobial agents in food preservation and potential biomedical applications. However, further research is necessary to elucidate the mechanisms of action, optimize synthesis methods, and assess the long-term effects and safety of AgNPs in different contexts.

While our study provides valuable insights into the efficacy of Silver Diamine Fluoride (SDF) on bacterial biofilms, there are some limitations to consider. The studies included in the analysis were limited to those published in English, which may introduce bias into the results. It is possible that studies conducted in other languages may have different findings, which were not included in the review. Moreover, the studies included in the analysis varied in their methodology, which may have influenced the results. Some studies had smaller sample sizes or shorter follow-up periods than others, which may have affected the overall conclusions drawn from the analysis. In addition, the review only included studies published up to a certain point in time, and new research may have been published since then that could affect the conclusions drawn from the analysis. It is also important to note that the review focused specifically on the effects of SDF on bacterial biofilms and did not examine other potential benefits or drawbacks of SDF treatment. Therefore, while the findings of the review are important for understanding the potential of SDF as a treatment for dental caries and related conditions, they do not provide a comprehensive analysis of the potential benefits and drawbacks of SDF treatment.

## 5. Conclusions

In conclusion, our systematic review and meta-analysis has revealed a very positive impact of SDF in comparison to other biomarkers mentioned in the 15 studies that were included in this review. The events favoring SDF exhibited statistical values which indicate that, on an overall basis, our systematic review and meta-analysis provide strong evidence that SDF is an effective treatment for reducing bacterial biofilms. This finding has important implications for improving oral health outcomes, particularly for individuals who may not have access to traditional dental treatments or who may have difficulty receiving such treatments. Given the clear correlation between the usage of SDF and the decrease in bacterial biofilms, we recommend that further research be conducted to explore the potential of SDF as a standard treatment option for dental caries and related conditions.

## Figures and Tables

**Table 1:** Search strategy.

*PubMed*: (“silver diamine fluoride” OR “SDF”) AND (“bacterial biofilms” OR “anti-bacterial agents”[MeSH Terms] OR “fluorides”[MeSH Terms] OR “dental caries”[MeSH Terms] OR “*Streptococcus mutans*”[MeSH Terms] OR “dental plaque”[MeSH Terms] OR “biofilms”[MeSH Terms]) AND English[lang]
*Web of Sciences:* (“silver diamine fluoride” OR “SDF”) AND (“bacterial biofilms” OR “anti-bacterial agents” OR “fluorides” OR “dental caries” OR “*Streptococcus mutans*” OR “dental plaque” OR “biofilms”) AND Language: (English)
*Scopus:* (“silver diamine fluoride” OR “SDF”) AND (“bacterial biofilms” OR “anti-bacterial agents” OR “fluorides” OR “dental caries” OR “*Streptococcus mutans*” OR “dental plaque” OR “biofilms”) AND (LIMIT-TO (DOCTYPE, “ar”) OR LIMIT-TO (DOCTYPE, “re”)) AND (LIMIT-TO (LANGUAGE, “English”))

**Table 2:** Variables selected and analysed for the review at the end of the data extraction protocol.

ID and Year	Study Sample (*n*)	Objectives	Design	Assessment Drawn
Bao Ying et al. (2020) [38]	Five children	To evaluate the antibacterial performance of SDF in dentine biofilms	In vitro	Microbial diversity fell after SDF application.
Ebtissam et al. (2019) [39]	Seventy dentin discs (made from extracted human teeth)	To evaluate the antibacterial performance of CHX and NaOCl as compared to SDF	Case control	SDF exhibited higher antibacterial efficacy as compared to the controls.
Gerd et al. (2017) [40]	100 samples of bovine dentin.	To evaluate the antibacterial performance of NaF and CHX as compared to SDF	In vitro	When compared to control, SDF dramatically reduced bacterial numbers.
Jaivrat et al. (2019) [41]	32 extracted human molars and 32 extracted human premolars	To evaluate the antibacterial performance of CHX, distilled water and PAA as compared to SDF	Case control	SDF-KI was deemed to be effective in eliminating *S. mutans*.
Kausar et al. (2020) [42]	35 *Candida* isolates	To evaluate the antifungal performance of SDF in isolation	In vitro	SDF appeared to successfully stop fungal filamentation even at extremely low doses, complementing its antibacterial activity
Klanliang et al. (2022) [43]	10 healthy individuals (aged between 26–31 years)	To evaluate the microbiological performance of SDF in dentine biofilms	In situ	Dental biofilm development was inhibited and the percentage of killed bacteria was enhanced when SDF was applied to demineralized dentin but only up to 4 days
Maribasappa et al. (2019) [44]	5 patients with carious lesions	To evaluate the antibacterial performance of SDF + potassium iodide (KI).	In vivo	In four of the five patients, SDF + KI totally stopped the growth of *S. mutans*
May L. Mei et al. (2014) [45]	12 primary upper-central carious incisors	To evaluate the physicochemical performance of SDF in carious teeth	Ex vivo	Clinical SDF application enhanced the levels of dentine remineralization
May Lei et al. (2016) [46]	6 premolars	To evaluate the antibacterial performance of SDF in two different types of restorations	In vitro	SDF application made both the types of restorations more resistant to subsequent caries.
Mitwalli et al. (2019) [47]	20 participants (who had at least one cervical carious lesion or soft cavitated root)	To evaluate the microbiological performance of SDF in carious lesions	In vitro	Several bacterium species showed a substantial decrease in relative abundance following SDF treatment.
Najmeh et al. (2018) [48]	45 extracted deciduous canines	To evaluate the antibacterial performance of fluorinated varnish as compared to SDF	In vitro	No significant differences between the antibacterial performance of both the compounds were observed
Parand Sorkhdini et al. (2020) [49]	90 human enamel samples	To evaluate the antibacterial performance of AgNO_3_, KF and water as compared to SDF	Case control	SDF performed on a similar parlance as DW and KF that SDF was compared with.
Rima et al. (2020) [50]	Samples of bovine dentin divided into 3 groups	To evaluate the antifungal performance of SDF in isolation	In vitro	SDF appeared to successfully stop fungal filamentation even at extremely low doses, complementing its antibacterial activity
Sunny et al. (2019) [51]	159 active dentinal carious lesions from primary molars	To evaluate the antibacterial performance of AgF as compared to SDF	RCT	SDF performed on a similar parlance as NaF that SDF was compared to
Vinson et al. (2018) [52]	*S. mutans* biofilm in six-well tissue culture plates	To evaluate the antibacterial performance of KI as compared to SDF	In vitro	SDF + KI performed with the highest efficacy, followerd by KI and SDF alone.

## Data Availability

The data presented in this study are available on request from the corresponding author. The data are not publicly available due to privacy related concerns.

## Abbreviations

SDF	Silver diamine fluoride
CHX	Chlorhexidine
HA	Hydroxyapatite
SDF-KI	Silver diamine fluoride + Potassium iodide
AgNPs	Silver nanoparticles
DW	Deionized water
PAA	Polyacrylic acid (PAA)

## References

[1] Marsh P.D., Moter A., Devine D.A. (2011). Dental plaque biofilms: Communities, conflict and control. Periodontol. 2000.

[2] Maiorana C., Beretta M., Grossi G.B., Santoro F., Herford A.S., Nagursky H., Cicciù M. (2011). Histomorphometric Evaluation of Anorganic Bovine Bone Coverage to Reduce Autogenous Grafts Resorption: Preliminary Results. Open Dent. J..

[3] Rengo C., Spagnuolo G., Ametrano G., Juloski J., Rengo S., Ferrari M. (2014). Micro-Computerized Tomographic Analysis of Premolars Restored with Oval and Circular Posts. Clin. Oral Investig..

[4] Cicciù M., Herford A.S., Stoffella E., Cervino G., Cicciù D. (2012). Protein-Signaled Guided Bone Regeneration Using Titanium Mesh and Rh-BMP2 in Oral Surgery: A Case Report Involving Left Mandibular Reconstruction after Tumor Resection. Open Dent. J..

[5] Mosaddad S.A., Tahmasebi E., Yazdanian A., Rezvani M.B., Seifalian A., Yazdanian M., Tebyanian H. (2019). Oral microbial biofilms: An update. Eur. J. Clin. Microbiol. Infect. Dis..

[6] Mira A., Simon-Soro A., Curtis M.A. (2017). Role of microbial communities in the pathogenesis of periodontal diseases and caries. J. Clin. Periodontol..

[7] Marsh P.D. (2003). Are dental diseases examples of ecological catastrophes?. Microbiology.

[8] Marsh P.D. (2006). Dental plaque as a biofilm and a microbial community—Implications for health and disease. BMC Oral Health.

[9] Marimallappa T.R., Pal S., Ashok K.K.R., Bhat P., Raghupathy R.K. (2021). Acomparative microbiological study of polyglycolic acid and silk sutures in oral surgical procedures. Minerva Dent. Oral Sci..

[10] Bowen W.H., Burne R.A., Wu H., Koo H. (2018). Oral Biofilms: Pathogens, Matrix, and Polymicrobial Interactions in Microenvironments. Trends Microbiol..

[11] Takahashi N., Nyvad B. (2016). Ecological Hypothesis of Dentin and Root Caries. Caries Res..

[12] Gati D., Vieira A.R. (2011). Elderly at Greater Risk for Root Caries: A Look at the Multifactorial Risks with Emphasis on Genetics Susceptibility. Int. J. Dent..

[13] Steele J.G., Sheiham A., Marcenes W., Fay N., Walls A.W.G. (2001). Clinical and behavioural risk indicators for root caries in older people. Gerodontology.

[14] Uccioli U., Fonzar A., Lanzuolo S., Meloni S.M., Lumbau A.I., Cicciù M., Tallarico M. (2021). Tissue Recession around a Dental Implant in Anterior Maxilla: How to Manage Soft Tissue When Things Go Wrong?. Prosthesis.

[15] Kleinberg I. (2002). A mixed-bacteria ecological approach to understanding the role of the oral bacteria in dental caries causation: An alternative to Streptococcus mutans and the specific-plaque hypothesis. Crit. Rev. Oral Biol. Med..

[16] Tanzer J.M., Livingston J., Thompson A.M. (2001). The Microbiology of Primary Dental Caries in Humans. J. Dent. Educ..

[17] Femiano F., Femiano R., Femiano L., Nucci L., Minervini G., Antonelli A., Bennardo F., Barone S., Scotti N., Sorice V. (2020). A New Combined Protocol to Treat the Dentin Hypersensitivity Associated with Non-Carious Cervical Lesions: A Randomized Controlled Trial. Appl. Sci..

[18] Hamada S., Slade H.D. (1980). Biology, immunology, and cariogenicity of Streptococcus mutans. Microbiol. Rev..

[19] Loesche W.J. (1986). Role of Streptococcus mutans in human dental decay. Microbiol. Rev..

[20] Peng J.-Y., Tsoi J.K.H., Matinlinna J.P., Botelho M.G. (2019). Silver deposition on demineralized dentine surface dosed by silver diammine fluoride with different saliva. J. Investig. Clin. Dent..

[21] Chu C.H., Lo E.C.M., Lin H.C. (2002). Effectiveness of Silver Diamine Fluoride and Sodium Fluoride Varnish in Arresting Dentin Caries in Chinese Pre-school Children. J. Dent. Res..

[22] Chu C.-H., Lee A.H.C., Zheng L., Mei M.L., Chan G.C.-F. (2014). Arresting rampant dental caries with silver diamine fluoride in a young teenager suffering from chronic oral graft versus host disease post-bone marrow transplantation: A case report. BMC Res. Notes.

[23] Amenta F., Battineni G., Kalyan L., Reddy V., Madithati P., Reddy Narapureddy B. (2022). Personalized Medicine Perception about Health Applications (Apps) in Smartphones towards Telemedicine during COVID-19: A Cross-Sectional Study. J. Pers. Med..

[24] Fiori A., Minervini G., Nucci L., d’Apuzzo F., Perillo L., Grassia V. (2022). Predictability of crowding resolution in clear aligner treatment. Prog. Orthod..

[25] Hendre A.D., Taylor G.W., Chávez E.M., Hyde S. (2017). A systematic review of silver diamine fluoride: Effectiveness and application in older adults. Gerodontology.

[26] Tan H.P., Lo E.C.M., Dyson J.E., Luo Y., Corbet E.F. (2010). A Randomized Trial on Root Caries Prevention in Elders. J. Dent. Res..

[27] Knight G.M., McIntyre J.M., Craig G.G., Mulyani Zilm P.S., Gully N.J. (2009). Inability to form a biofilm of Streptococcus mutans on silver fluoride—And potassium iodide-treated demineralized dentin. Quintessence Int..

[28] Mei M.L., Ito L., Cao Y., Li Q.L., Lo E.C.M., Chu C.H. (2013). Inhibitory effect of silver diamine fluoride on dentine demineralisation and collagen degradation. J. Dent..

[29] Chu C.H., Mei L.E.I., Seneviratne C.J., Lo E.C.M. (2012). Effects of silver diamine fluoride on dentine carious lesions induced by Streptococcus mutans and Actinomyces naeslundii biofilms. Int. J. Paediatr. Dent..

[30] Mei M.L., Nudelman F., Marzec B., Walker J.M., Lo E.C.M., Walls A.W., Chu C.H. (2017). Formation of Fluorohydroxyapatite with Silver Diamine Fluoride. J. Dent. Res..

[31] Knight G., McIntyre J., Craig G., Mulyani Zilm P., Gully N. (2005). An in vitro model to measure the effect of a silver fluoride and potassium iodide treatment on the permeability of demineralized dentine to Streptococcus mutans. Aust. Dent. J..

[32] Mei M.L., Li Q., Chu C.-H., Lo E.-M., Samaranayake L.P. (2013). Antibacterial effects of silver diamine fluoride on multi-species cariogenic biofilm on caries. Ann. Clin. Microbiol. Antimicrob..

[33] Mei M.L., Chu C.H., Low K.H., Che C.M., Lo E.C.M. (2013). Caries arresting effect of silver diamine fluoride on dentine carious lesion with S. mutans and L. acidophilus dual-species cariogenic biofilm. Med. Oral Patol. Oral Cir. Bucal..

[34] Minervini G., Franco R., Marrapodi M.M., Mehta V., Fiorillo L., Badnjević A., Cervino G., Cicciù M. (2023). The Association between COVID-19 Related Anxiety, Stress, Depression, Temporomandibular Disorders, and Headaches from Childhood to Adulthood: A Systematic Review. Brain Sci.

[35] Qamar Z., Alghamdi A.M.S., Bin Haydarah N.K., Balateef A.A., Alamoudi A.A., Abumismar M.A., Shivakumar S., Cicciù M., Minervini G. (2023). Impact of Temporomandibular Disorders on Oral Health Related Quality of Life: A Systematic Review and Meta-analysis. J. Oral Rehabil..

[36] Watson P.S., Pontefract H.A., Devine D.A., Shore R.C., Nattress B.R., Kirkham J., Robinson C. (2005). Penetration of Fluoride into Natural Plaque Biofilms. J. Dent. Res..

[37] Prada-López I., Quintas V., Vilaboa C., Suárez-Quintanilla D., Tomás I. (2016). Devices for In situ Development of Non-disturbed Oral Biofilm. A Systematic Review. Front Microbiol..

[38] Liu B.-Y., Liu J., Zhang D., Yang Z.-L., Feng Y.-P., Wang M. (2020). Effect of silver diammine fluoride on micro-ecology of plaque from extensive caries of deciduous teeth—In vitro study. BMC Oral Health.

[39] Al-Madi E.M., Al-Jamie M.A., Al-Owaid N.M., Almohaimede A.A., Al-Owid A.M. (2019). Antibacterial efficacy of silver diamine fluoride as a root canal irrigant. Clin. Exp. Dent. Res..

[40] Göstemeyer G., Schulze F., Paris S., Schwendicke F. (2017). Arrest of Root Carious Lesions via Sodium Fluoride, Chlorhexidine and Silver Diamine Fluoride In Vitro. Materials.

[41] Gupta J., Thomas M., Radhakrishna M., Srikant N., Ginjupalli K. (2019). Effect of silver diamine fluoride-potassium iodide and 2% chlorhexidine gluconate cavity cleansers on the bond strength and microleakage of resin-modified glass ionomer cement. J. Conserv. Dent..

[42] Fakhruddin K.S., Egusa H., Ngo H.C., Panduwawala C., Pesee S., Venkatachalam T., Samaranayake L.P. (2020). Silver diamine fluoride (SDF) used in childhood caries management has potent antifungal activity against oral Candida species. BMC Microbiol..

[43] Klanliang K., Asahi Y., Maezono H., Sotozono M., Kuriki N., Machi H., Ebisu S., Hayashi M. (2022). An extensive description of the microbiological effects of silver diamine fluoride on dental biofilms using an oral in situ model. Sci. Rep..

[44] Karched M., Ali D., Ngo H. (2019). In vivo antimicrobial activity of silver diammine fluoride on carious lesions in dentin. J. Oral Sci..

[45] Mei M.L., Ito L., Cao Y., Lo E.C.M., Li Q.L., Chu C.H. (2014). An ex vivo study of arrested primary teeth caries with silver diamine fluoride therapy. J. Dent..

[46] Mei M.L., Zhao I.S., Ito L., Lo E.C.-M., Chu C.-H. (2016). Prevention of secondary caries by silver diamine fluoride. Int. Dent. J..

[47] Mitwalli H., Mourao M.D.A., Dennison J., Yaman P., Paster B.J., Fontana M. (2019). Effect of Silver Diamine Fluoride Treatment on Microbial Profiles of Plaque Biofilms from Root/Cervical Caries Lesions. Caries Res..

[48] Mohammadi N., Farahmand Far M. (2018). Effect of fluoridated varnish and silver diamine fluoride on enamel demineralization resistance in primary dentition. J. Indian Soc. Pedod. Prev. Dent..

[49] Sorkhdini P., Gregory R.L., Crystal Y.O., Tang Q., Lippert F. (2020). Effectiveness of in vitro primary coronal caries prevention with silver diamine fluoride—Chemical vs biofilm models. J. Dent..

[50] Alshahni R.Z., Alshahni M.M., Hiraishi N., Makimura K., Tagami J. (2020). Effect of Silver Diamine Fluoride on Reducing Candida albicans Adhesion on Dentine. Mycopathologia.

[51] Tirupathi S., Nirmala S.V.S.G., Rajasekhar S., Nuvvula S. (2019). Comparative cariostatic efficacy of a novel Nano-silver fluoride varnish with 38% silver diamine fluoride varnish a double-blind randomized clinical trial. J. Clin. Exp. Dent..

[52] Vinson L.A., Gilbert P.R., Sanders B.J., Moser E., Gregory R.L. (2018). Silver Diamine Fluoride and Potassium Iodide Disruption of In Vitro Streptococcus mutans Biofilm. J. Dent. Child..

[53] Li R., Lo E.C.M., Liu B.Y., Wong M.C.M., Chu C.H. (2016). Randomized clinical trial on arresting dental root caries through silver diammine fluoride applications in community-dwelling elders. J. Dent..

[54] Wierichs R.J., Meyer-Lueckel H. (2015). Systematic Review on Noninvasive Treatment of Root Caries Lesions. J. Dent. Res..

[55] Duangthip D., Chu C.H., Lo E.C.M. (2016). A randomized clinical trial on arresting dentine caries in preschool children by topical fluorides—18 month results. J. Dent..

[56] Llodra J.C., Rodriguez A., Ferrer B., Menardia V., Ramos T., Morato M. (2005). Efficacy of Silver Diamine Fluoride for Caries Reduction in Primary Teeth and First Permanent Molars of Schoolchildren: 36-month Clinical Trial. J. Dent. Res..

[57] Lau N., O’Daffer A., Yi-Frazier J.P., Rosenberg A.R. (2021). Popular Evidence-Based Commercial Mental Health Apps: Analysis of Engagement, Functionality, Aesthetics, and Information Quality. JMIR Mhealth Uhealth.

[58] Greenwall-Cohen J., Greenwall L., Barry S. (2020). Silver diamine fluoride—An overview of the literature and current clinical techniques. Br. Dent. J..

[59] Al Qranei M.S., Balhaddad A.A., Melo M.A.S. (2021). The burden of root caries: Updated perspectives and advances on management strategies. Gerodontology.

[60] Jiang M., Mei M.L., Wong M.C.M., Chu C.H., Lo E.C.M. (2020). Effect of silver diamine fluoride solution application on the bond strength of dentine to adhesives and to glass ionomer cements: A systematic review. BMC Oral Health.

[61] Jiang M., Wong M.C.M., Chu C.H., Dai L., Lo E.C.M. (2019). Effects of restoring SDF-treated and untreated dentine caries lesions on parental satisfaction and oral health related quality of life of preschool children. J. Dent..

[62] Knight G., McIntyre J., Craig G., Zilm P., Gully N. (2007). Differences between normal and demineralized dentine pretreated with silver fluoride and potassium iodide after an in vitro challenge by Streptococcus mutans. Aust. Dent. J..

[63] Sayed M., Matsui N., Hiraishi N., Nikaido T., Burrow M., Tagami J. (2018). Effect of Glutathione Biomolecule on Tooth Discoloration Associated with Silver Diammine Fluoride. Int. J. Mol. Sci..

[64] Sayed M., Hiraishi N., Matin K., Abdou A., Burrow M.F., Tagami J. (2020). Effect of silver-containing agents on the ultra-structural morphology of dentinal collagen. Dent. Mater..

[65] Schwass D.R., Lyons K.M., Love R., Tompkins G.R., Meledandri C.J. (2018). Antimicrobial Activity of a Colloidal AgNP Suspension Demonstrated In Vitro against Monoculture Biofilms: Toward a Novel Tooth Disinfectant for Treating Dental Caries. Adv. Dent. Res..

[66] Ramazanli V.N., Ahmadov I.S. (2022). Synthesis of Silver Nanoparticles by Using Extract of Olive Leaves. Adv. Biol. Earth Sci..

[67] Baran A., Fırat Baran M., Keskin C., Hatipoğlu A., Yavuz Ö., İrtegün Kandemir S., Eftekhari A. (2022). Investigation of antimicrobial and cytotoxic properties and specification of silver nanoparticles (AgNPs) derived from *Cicer arietinum* L. green leaf extract. Front. Bioeng. Biotechnol..

